# A high-throughput imaging and quantification pipeline for the EVOS imaging platform

**DOI:** 10.1371/journal.pone.0236397

**Published:** 2020-08-05

**Authors:** Stefan Donovan Klimaj, Yamhilette Licon Munoz, Katelyn Del Toro, William Curtis Hines

**Affiliations:** Department of Biochemistry and Molecular Biology, University of New Mexico School of Medicine, Albuquerque, New Mexico, United States of America; Baylor College of Medicine, UNITED STATES

## Abstract

Self-contained imaging systems are versatile instruments that are becoming a staple in cell culture laboratories. Many of these machines possess motorized stages and on-stage incubators that permit programmable imaging of live cells that make them a sensible tool for high-throughput applications. The EVOS imaging system is such a device and is capable of scanning multi-well dishes and stitching together multiple adjacent fields to produce coherent individual images of each well. Automated batch analysis and quantification of these tiled images does however require off-loading files to other software platforms. Our initial attempts to quantify tiled images captured on an EVOS device was plagued by some expected—and other unforeseeable—issues that arose at nearly every stage of analysis. These included: high background, illumination and stitching artifacts, low contrast, noise, focus inconsistencies, and image distortion—all of which negatively impacted processing efficiency. We have since overcome these obstacles and have created a rigorous cell counting pipeline for analyzing images captured by the EVOS scan function. We present development and optimization of this automated pipeline and submit it as an effective and facile tool for accurately counting cells from tiled images.

## Introduction

Miniaturization of advanced microscopy equipment and automation of data collection now permit acquisition of high-quality fluorescent images within the confines of a typical cell laboratory. Accessibility to these systems has increased the sheer volume of images that can be generated in short time, yielding densely layered data that becomes unwieldly without assistance of computational algorithms. The EVOS Automated Cell Imaging System (ThermoFisher Scientific) is such a device, and we frequently use our machine for high-throughput imaging applications and experiments that require quantifying absolute numbers of cells in multi-well dishes. Other common methods and endpoint assays of cell quantification in these types of experiments are possible; e.g., MTT or ATP assays, but quantification afforded by imaging is non-destructive and allows an accurate and dynamic assessment of cell-division over the course of a month’s-long experiment.

A common practice when scanning multi-well dishes is to image random fields, which produces results that are relative. However, there are instances where using random fields for quantification may be confounding. For example, we frequently perform experiments where we deposit a limiting number of cells (e.g., 1,10, or 50) into individual wells of a 96-well culture plate. In this case, cells occupy only a small fraction of the available growth area, so it’s essential the entire well be imaged to allow for an accurate count. Because an entire well cannot fit into a single microscopic field, imaging multiple overlapping fields is required.

The EVOS imaging system contains a scanning algorithm that screens culture plates and creates individual tiled images of each well. However, high-throughput analysis of these mosaic images requires use of other software platforms for quantification, such as: CellProfiler [1, 2], ImageJ [3], or Matlab/Octave [4, 5]. These software programs are powerful tools, but they do have many parameters that can be applied and/or adjusted. Finding the ideal combination of these features—that avoids artifacts and produces accurate cell counts—often requires a significant investment of time.

The substantial effort required to manually analyze images captured in our experiments was causing significant delays, so we sought to develop an automated pipeline that could quickly and rigorously count cells cultured in 96-well dishes (from stitched images). Through this exercise, we encountered multiple problems that severely affected results (Table 1)—not all of which we predicted from the outset. Here, we present these problems, as well as solutions to each. We describe an analytical pipeline for quantifying cells within stitched images generated by the EVOS imaging system—a method that requires little user input and can be easily adopted for high throughput screening assays.

**Table 1:** Problems encountered during pipeline development.

	Problem	Cause	Solution	Fig
IMAGING	High Background (autofluorescence)	Growth medium	Use imaging DMEM or PBS*	2
	Image noise	Impurities in medium/serum	Prefilter medium (0.2 um); apply gaussian blur (step 12)	3
	Long scan times	Autofocus	Use manual settings	5
	Image brightness varies	Autoexposure	Use manual settings	4
	Distorted ovoid images	Insufficient virtual memory	Increase memory allocation	6
PROCESSING	Position of well varies (in tiled images)	Stitching; Evos software	Identify well as object; apply mask (steps 1–6)	7
	EVOS Stitching artifacts	Illumination artifact compounded by tiling	Apply illumination correction (steps 9–10)	SF1
	Dim cells after illumination correction, (steps 7–8)	Illumination correction reduces contrast	Log-contrast transformation (steps 7–8)	SF1
	Edge of well interpreted as cells	Applied mask didn’t remove the entire well	Contract the mask (erosion, step 11)	SF1
	Too many cells being detected by CellProfiler	Imperfect threshold setting	Manually adjust (optimize) threshold setting (step 13)	SF1

*Note that PBS is not recommended for long scan times

## Methods

### Cell lines

MDA-MB-231 and MCF-7 breast cancer cell lines were obtained directly from the American Type Culture Collection (ATCC) and were cultured in high glucose Dulbecco’s modified eagle’s medium (DMEM, Sigma), supplemented with 10% FBS and 1x penicillin/streptomycin, as reported previously [6]. Defined cell numbers were deposited into 96 multi-well dishes using a Sony iCyt SY3200 flow sorter. Plates were cultured at 37°C in a humidified incubator (5% CO_2_) and were fed/imaged weekly. Media used in the imaging experiments include: Imaging DMEM (Fluorobrite DMEM, Gibco), DMEM/F12 (Thermo), and custom breast cell medium M87 [7], as indicated in figure legends.

### Cell imaging

Multi-well culture plates were imaged using an EVOS FL Auto imaging system equipped with an optional On-Stage-Incubator (OSI) that permitted control of temperature, humidity and CO_2_ levels during image acquisition. All imaging was performed at 4x objective magnification (4x PLAN/0.13NA long working distance objective) using the EVOS software’s ‘Scan’ function. H2B-GFP was imaged with the ‘GFP’ LED filter cube (470/22 excitation; 510/42 emission). To capture the entirety of each well, we set the machine to scan ‘Center Region Only’, typically at a setting of 75% or higher that guided the system to capture adjacent images roughly centered on each well, creating a 3x3 or 4x3 tiled image for each at these settings. It is essential that manual exposure (‘Actual’ mode) is used to acquire images to restrict the EVOS system’s autonomy for varying exposure time. At the end of the scan, the software produces 96 stitched image files in a lossless format (*.PNG) that were saved and used for analysis and cell quantification.

### Software

Software packages used in this work include CellProfiler 3.1.9 [1, 2], ImageJ [3], and Matlab R2018b (Mathworks) [4] (or GNU Octave [5] -an open-source Matlab alternative). The bulk of processing is performed in CellProfiler, and our final pipeline is supplied in the S1 Fig that can be found also at https://cellprofiler.org/examples/published_pipelines.

## Results

### Experimental parameters for acquiring high contrast images

**Fig 1:**
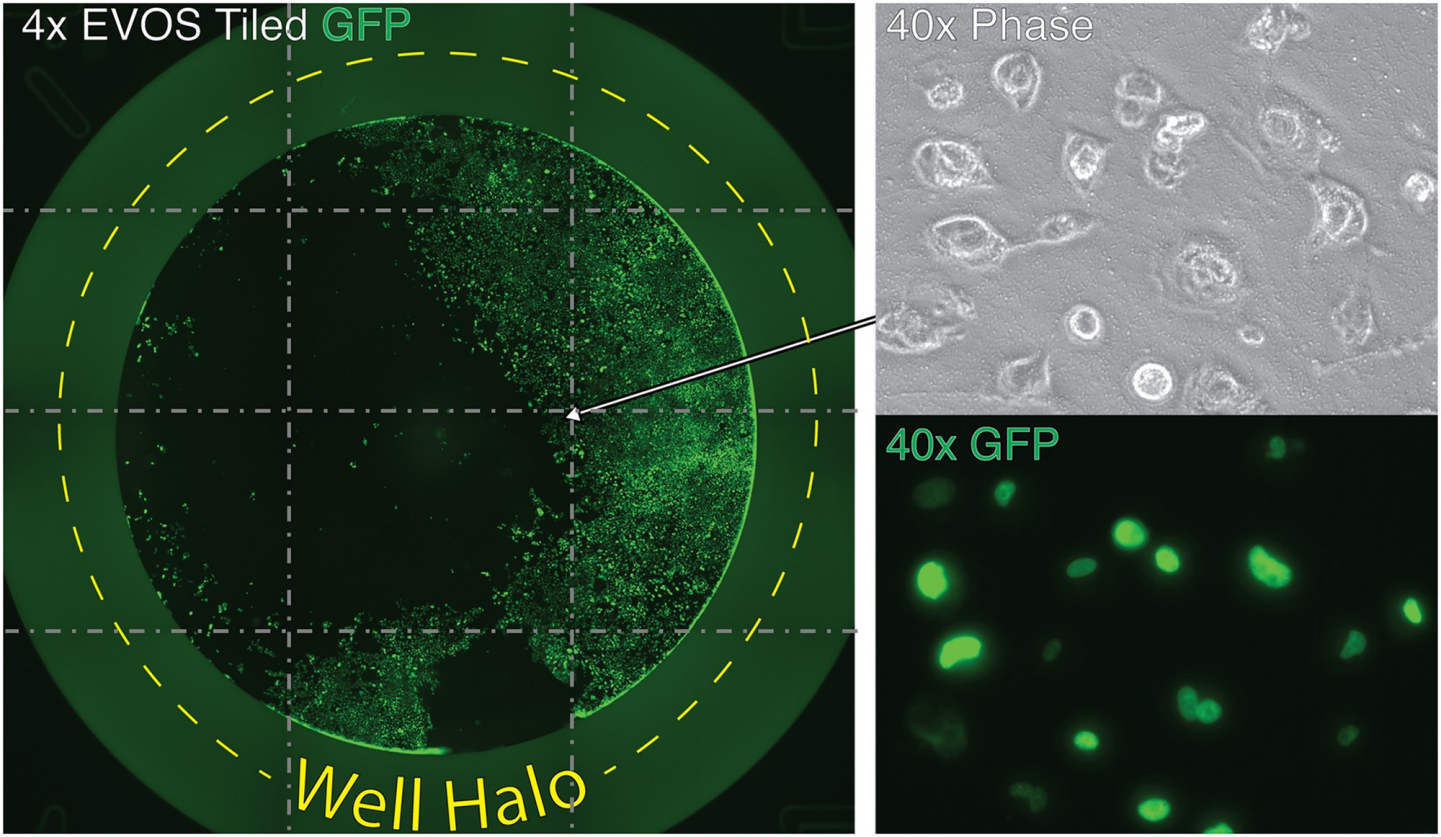
Mosaic image generated by EVOS. On left, is a 4x tiled scan (a composite of 4x3 images) of a single well of a 96-well plate containing H2B-GFP labeled MCF-7 cancer cells. We have overlaid a gray dashed line to indicate the edge of each image. A 40x phase contrast image of these cells is displayed in the upper right panel, whereas the fluorescent GFP channel is provided at bottom right. The perimeter of the well appears as a bright green fluorescent, labeled ‘well halo’ (indicated by the yellow dashed line).

To determine the effects of growth factors (or other media components) on cell proliferation, we often seed cells into 96-well dishes at clonal densities—reserving several rows for multiple biological replicates. For the purpose of developing our imaging pipeline, we used MCF-7 and MDA-MB-231 breast cancer cell lines labeled with histone-2-beta green-fluorescent-protein (H2B-GFP) [6, 8]. We used H2B-GFP because it causes the cell’s nuclei to fluoresce and helps the computer identify and distinguish between neighboring cells. Growth was assessed weekly by imaging the plates using the ‘Scan’ function of the EVOS FL Auto imaging system. This function directs the microscope to capture neighboring microscopic fields at each well location, and combines neighboring images together to create a composite, ‘tiled,’ image of each well. A 4x3 tiled image is illustrated in Fig 1. The composite images that are produced contain a central circular growth area encircled by a bright fluorescent ‘halo’ (produced by the perimeter of each well). At 4x magnification, we’ve found an entire well (of a 96-well dish, 0.32 cm^2^) can be captured in nine photographs (3x3 tiled image) with enough resolution to resolve cells and permit cell counting (not shown). After imaging, counting an entire set of images from a dish (96 wells) was consistently taking us between 7–8 hours to complete in ImageJ, largely because of the manual cropping required. Because of this analysis bottleneck, we decided to develop an automated pipeline to accelerate the process. We chose to use CellProfiler^™^ imaging software because it is open-source, intuitive, and feature rich. To facilitate automated counting, we needed to first optimize experimental conditions to create high quality images of uniform intensity and high contrast.

**Fig 2:**
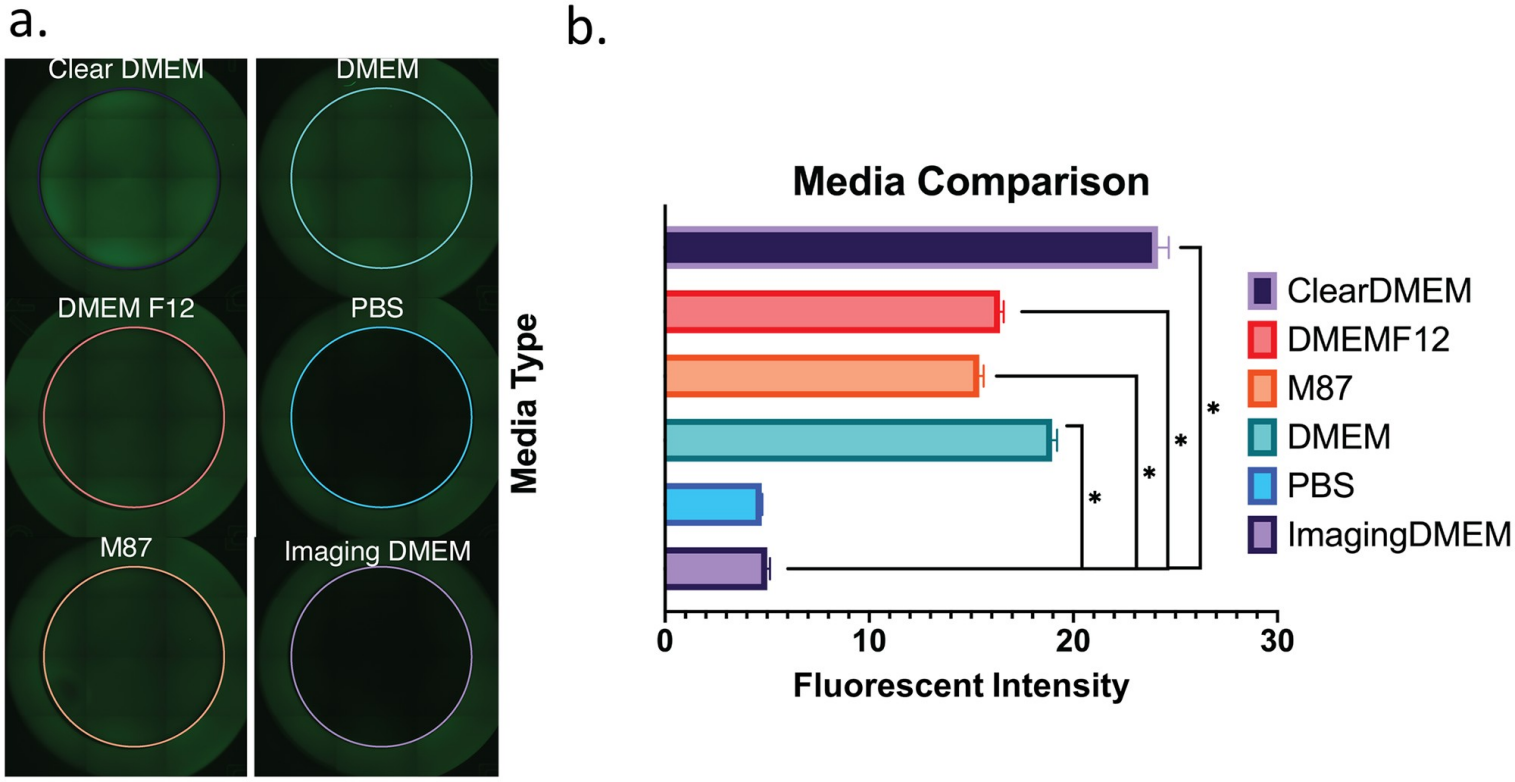
Fluorescence produced by cell media. a) Representative tiled-images of wells containing different media (phenol red-free ‘ClearDMEM;’ standard ‘DMEM’ containing phenol red; ‘DMEM/F12;’ phosphate buffered saline ‘PBS;’ ‘‘M87’ breast epithelial medium; and FlouroBrite^™^ ‘Imaging DMEM’ b) Fluorescent intensity within the colored circles (shown in 2a) was measured by ImageJ (n = 6 for each condition). * statistically significant (p<0.05, t-student test).

As anticipated, the first problem we encountered was the need for a different imaging medium. Our standard DMEM produced high levels of fluorescence background, likely from the vitamins and phenol red in the medium, as phenol red absorbs light across a broad spectrum (400-500nm at pH 7) [9] overlapping with GFP [10]. To circumvent this, a simple solution was to replace the DMEM with phosphate buffered saline (PBS). This worked well, however it’s not unexpected that the cells didn’t tolerate being in PBS over long periods—especially the hour or more required to image an entire plate. Seeking an alternative solution, we measured the background fluorescence generated by four different types of media (Fig 2). These included a phenol-red free DMEM (ClearDMEM), DMEM/F12, M87 (specific for mammary epithelial cells), and a medium designed specifically for fluorescent imaging (FluoroBrite^™^ DMEM, Thermo). Not surprisingly, the DMEM designed for fluorescent imaging performed the best, generating minimal background fluorescence that was roughly equivalent to PBS (Fig 2). Based on these results, we performed all future cell imaging experiments using FluoroBrite^™^ imaging DMEM.

**Fig 3:**
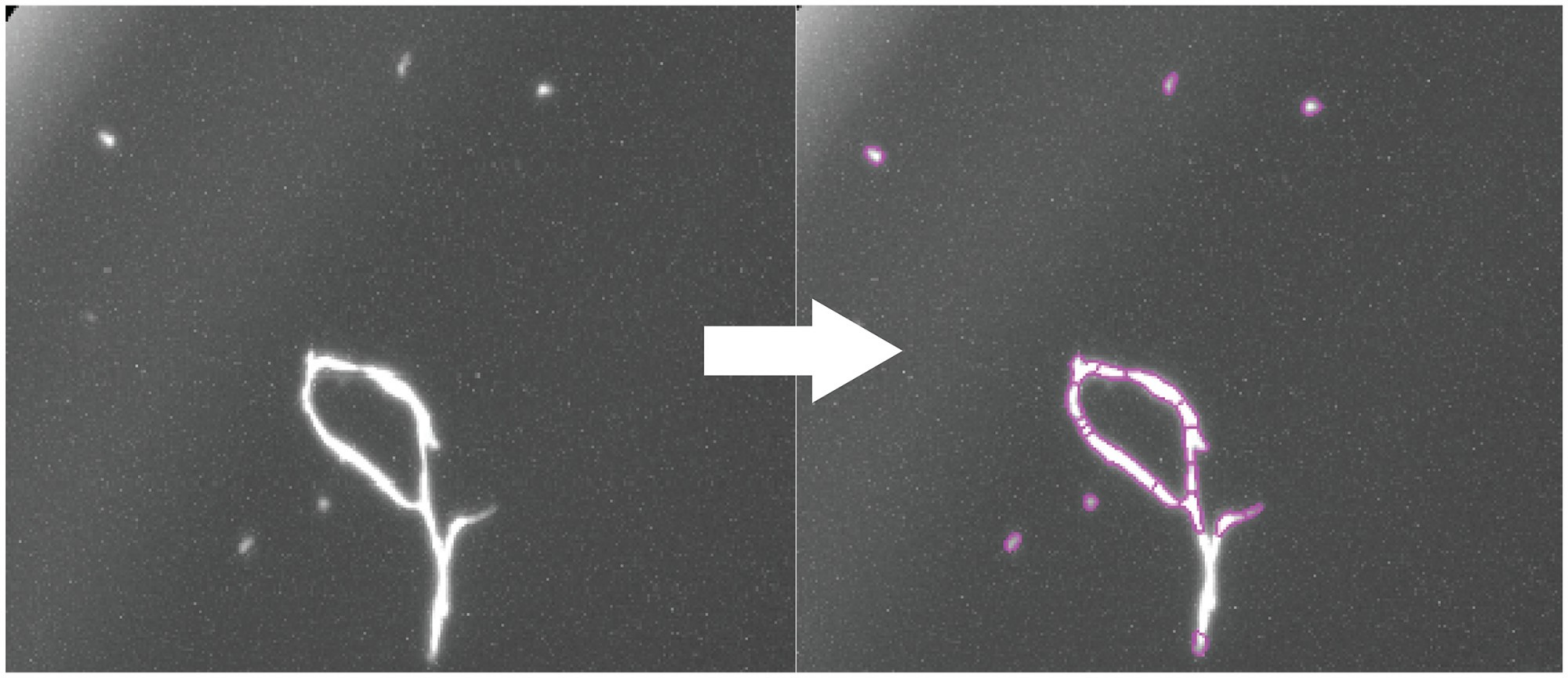
Debris in unfiltered medium interferes with cell counts. Unfiltered DMEM (supplemented with 10% FBS) contains particulates that the imaging software will incorrectly identify as cells (purple outlines).

The complete medium we use for these cells is supplemented with fetal bovine serum (FBS). Unfortunately, adding FBS to the medium did introduce insoluble particulates that interfered with image processing (Fig 3). To remove these particulates, we it was crucial we filter the FBS-supplemented FluoroBrite medium through a 0.2um filtration unit. We found the cell lines tolerated the imaging DMEM well, and it led to acquisition of high contrast images with readily detectable H2B-GFP+ cells.

**Fig 4:**
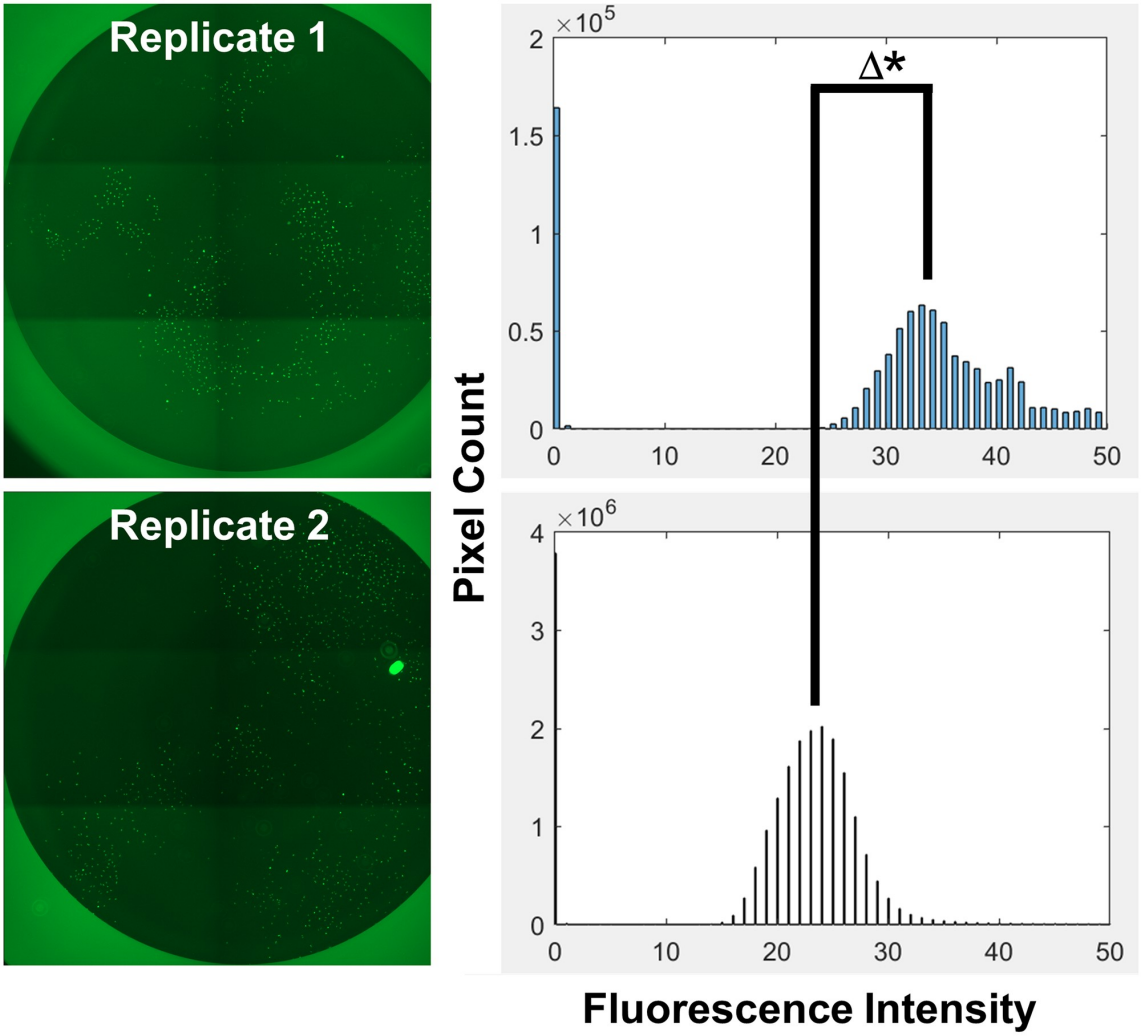
Luminosity variance of EVOS autoexposure. On left, two replicate wells captured using EVOS’ autoexposure setting. On right, Matlab histogram analysis of pixel count and fluorescence intensity of these two replicates. Δ symbol emphasizes difference in mean fluorescence intensities between replicates (introduced by the automatic exposure setting). * p<0.0001 (t-test of random background fields (free of cells), n = 18 fields for each replicate).

Although imaging DMEM drastically reduced background fluorescence, we noticed that there was still noticeable variation in image brightness between wells—even in replicate wells with identical treatments (Fig 4). We traced this problem to the autoexposure setting in the EVOS software and found this could be prevented if we set the exposure manually, using the software’s ‘Actual’ mode. Using this manual mode indeed created tiled images of consistent brightness that, in turn, was critical for CellProfiler to batch process and detect GFP positive cells. With the brightness inconsistencies solved, our next goal was to reduce the scan time for each 96-well plate, which at the time was taking several hours.

**Fig 5:**
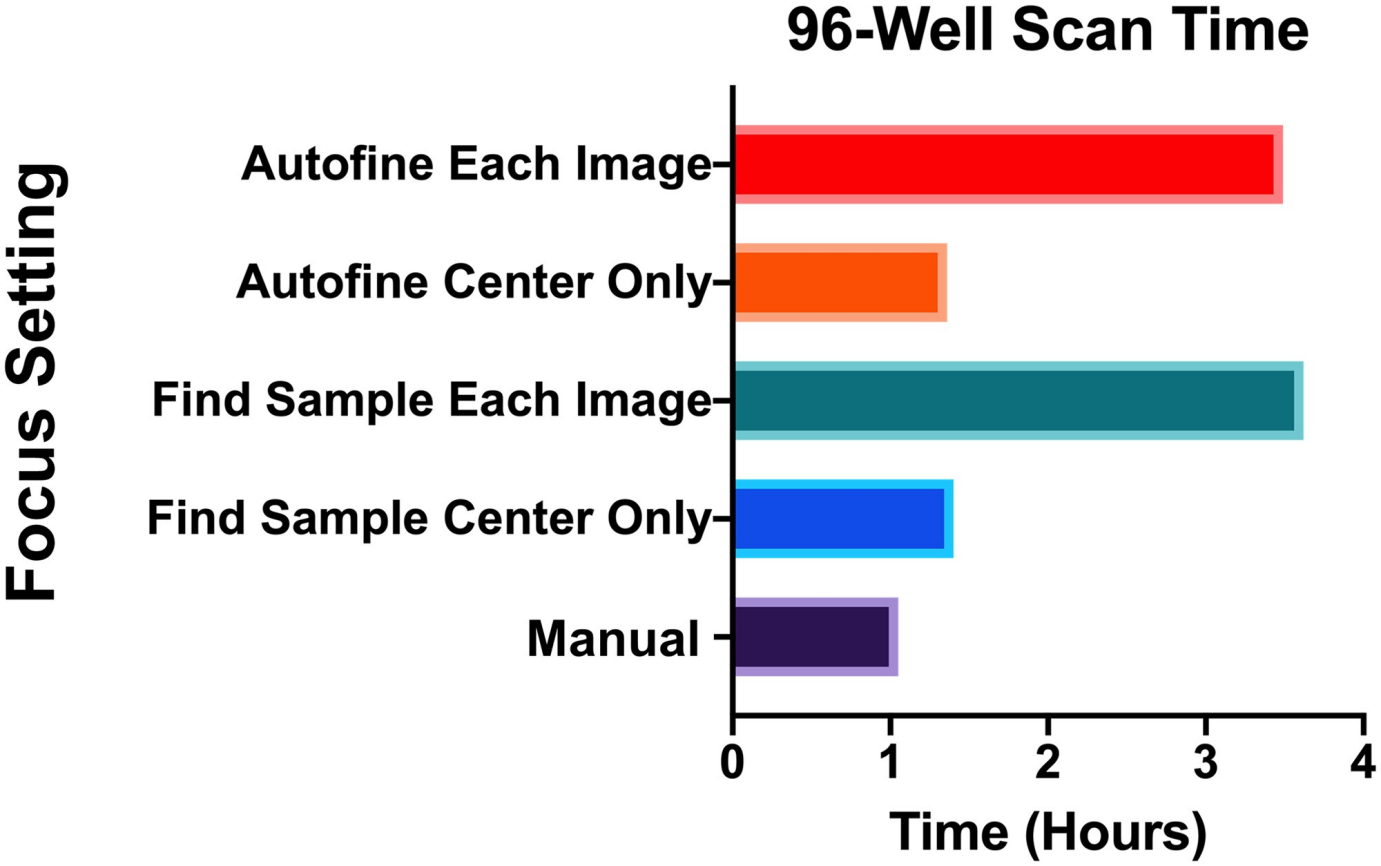
The impact of autofocus on scan time. The time to scan an entire plate varies among the five EVOS focus settings (Autofine each image, Autofine center only, Find sample each image, Find sample center only, and Manual).

In early experiments, we had EVOS autofocus the microscope for each image, which indeed produced crisp images. However, it expectedly took the machine a considerable amount of time to adjust and find the correct focal plane. This was compounded when an imaging field contained few or no cells, causing the machine to search repeatedly for the correct focal plane. Because we seed cells at low density in our experiments, this was the case most of the time. To determine if we could accelerate the imaging process, we deactivated the autofocus function. To help set the correct focal plane, we seeded the corner wells with extra cells (~500) and manually focused on these. There is some slight variance in the thickness of the plates, so perfectly focused images were not expected. However, we were able to set a compromising focal plane that produced acceptable images for cell identification. Using manual focus in this way reduced the scan time by more than two hours, meaning a single 96-well plate could now be imaged in just over one hour (Fig 5). With this and the above parameters set, we were ready to begin analyzing images.

**Fig 6:**
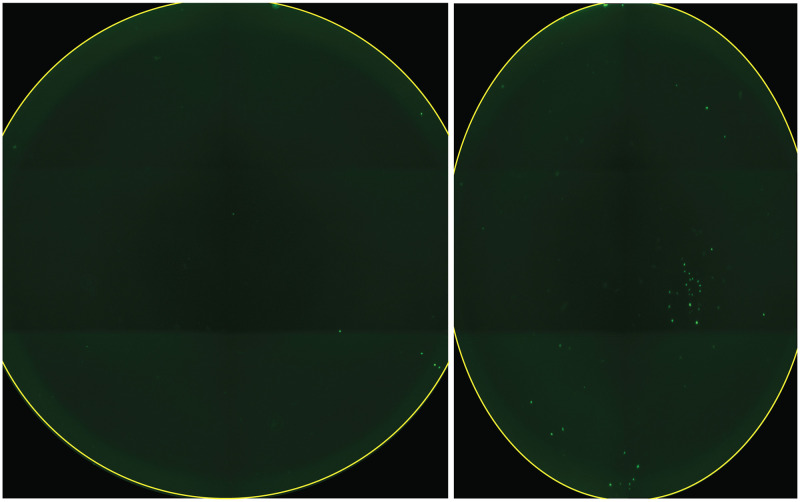
Compression error caused by software settings. On left, an image of a 96-well produced by EVOS. On right, a saved image of a well (from the same plate/scan) that has compressed the well into an ovoid shape. This problem was corrected by increasing the computer’s virtual memory allocation.

As we began loading images into CellProfiler, we discovered a curious problem: a few of the acquired images were compressed along the y-axis, transforming the well into an ovoid shape (Fig 6). This was puzzling, and occurred only when we imaged an entire plate, making it that much more difficult to diagnose. With the help of Thermo Technical Services, we eventually traced this problem to the virtual memory of the PC controlling the EVOS system. Increasing the operating system’s virtual memory allocation alleviated the problem.

### Development of the analysis pipeline

**Fig 7:**
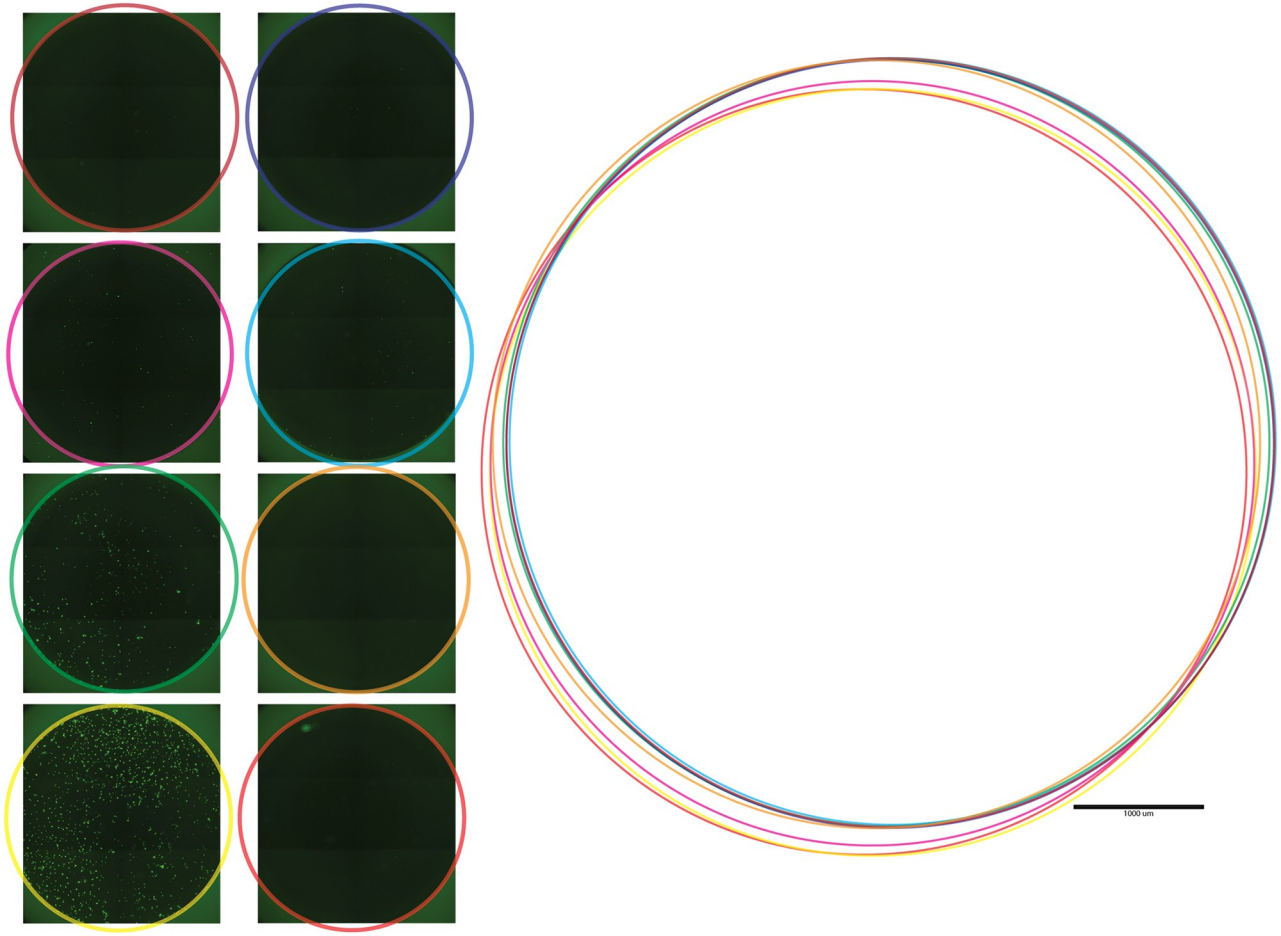
Well location variability. Comparing well A1 from eight 96-well plates shows the variability of the well location in the respective images. The growth area of each well is indicated by the color outlines (left). When these are overlaid, the variability is evident (right).

Having optimized culture conditions (medium), computer settings (virtual memory allocation), and EVOS acquisition options (4x objective, focus and exposure settings), we were confident that the acquired tiled images were ready to be analyzed by CellProfiler. Our first objective was to isolate the growth area by removing the fluorescent border produced by the plastic wall that circumscribes each well (‘well halo,’ Fig 1). Our original plan was to create a mask that could be applied universally to each image, but we discovered the precise location of the well (within each image) varied across the 96 acquired images (Fig 7). One solution we considered was to create cropping masks unique to each well position. However, when we created these masks, we found the positions of wells across different plates was also not consistent. Applying these masks was removing significant portions of the well and often left edges that interfered with analysis. Another option we explored was to create a uniform mask that would be small enough to fit inside the well area and not intersect with any of the variable well positions (Fig 7). This however would result in a loss of more than 25% of the growth area of each well. We deemed this unacceptable because it ran contrary to our main goal of imaging every cell in the well. We thus needed a creative solution to crop the well’s edge and extract the growth area within each image.

We found manually cropping the images in ImageJ [11] (shift+oval selection for circular ROI, edit > selection > make inverse, delete selection) produced accurate results. However, it took over an hour to manually crop an entire plates’ worth of images, so this was not practical. We ultimately found a viable solution in CellProfiler, via the ‘Identify Primary Objects’ module. We discovered that if we treated the well halo as a single object of interest (much like we do when we identify cells), it would direct the program to isolate the well no matter its position in the image. The trick was to direct the software to look for a very large object. This strategy worked and allowed us to create a template mask for each image, allowing the exterior portion of the well to be removed. Applying this function to all 96 wells took the software about 15 minutes to complete, and this became our first processing step after image acquisition (S1 Fig, Steps 1–6). Standard techniques were then applied to advance the pipeline towards automated cell detection. These steps included converting the color image to greyscale, inverting the image, applying a gaussian blur to smoothen edges, and contracting the well mask slightly (to prevent potential perturbances along the edge of the well from being incorrectly identified as cells, Fig 7). This flexible approach and combination of settings prepared the images for cell detection, but there was another unique problem that we’d need to overcome: an illumination artifact caused by the stitching algorithm.

Although the EVOS’ tiling function produces a composite image of each well, the brightness in the contributing images is not homogeneous. The center is brightest and dims towards the edges. This lighting artifact is common to fluorescent microscopy, and CellProfiler does contain an algorithm to remove this radial illumination artifact from single fields. However, it cannot be used on tiled images. These composite images have a soft grid pattern due to the lighting artifact (this can be observed in Figs 1, 2 and 4). Because the brightness of the grid’s edges often approached that of cells, the software would repeatedly incorrectly detect the grid as many cells, skewing cell counts dramatically. We thus needed a strategy to attenuate the grid. This was eventually accomplished by calculating an illumination function for the image set, which we then applied to each image to correct the uneven lighting. An unexpected benefit of this approach is that it also removed condensation artifacts that would sometimes be misidentified as cell (these were bright areas due to condensation on the plate’s cover). The precise settings we used for this (along with all other steps) are provided in the supplemental materials (CellProfiler Pipeline). Although applying this function to the images did remove the grid, it unfortunately also dimmed the H2B-GFP signal, causing the software to overlook large numbers of cells. To overcome this problem, we needed a strategy to enhance the GFP cell signal.

**Fig 8:**
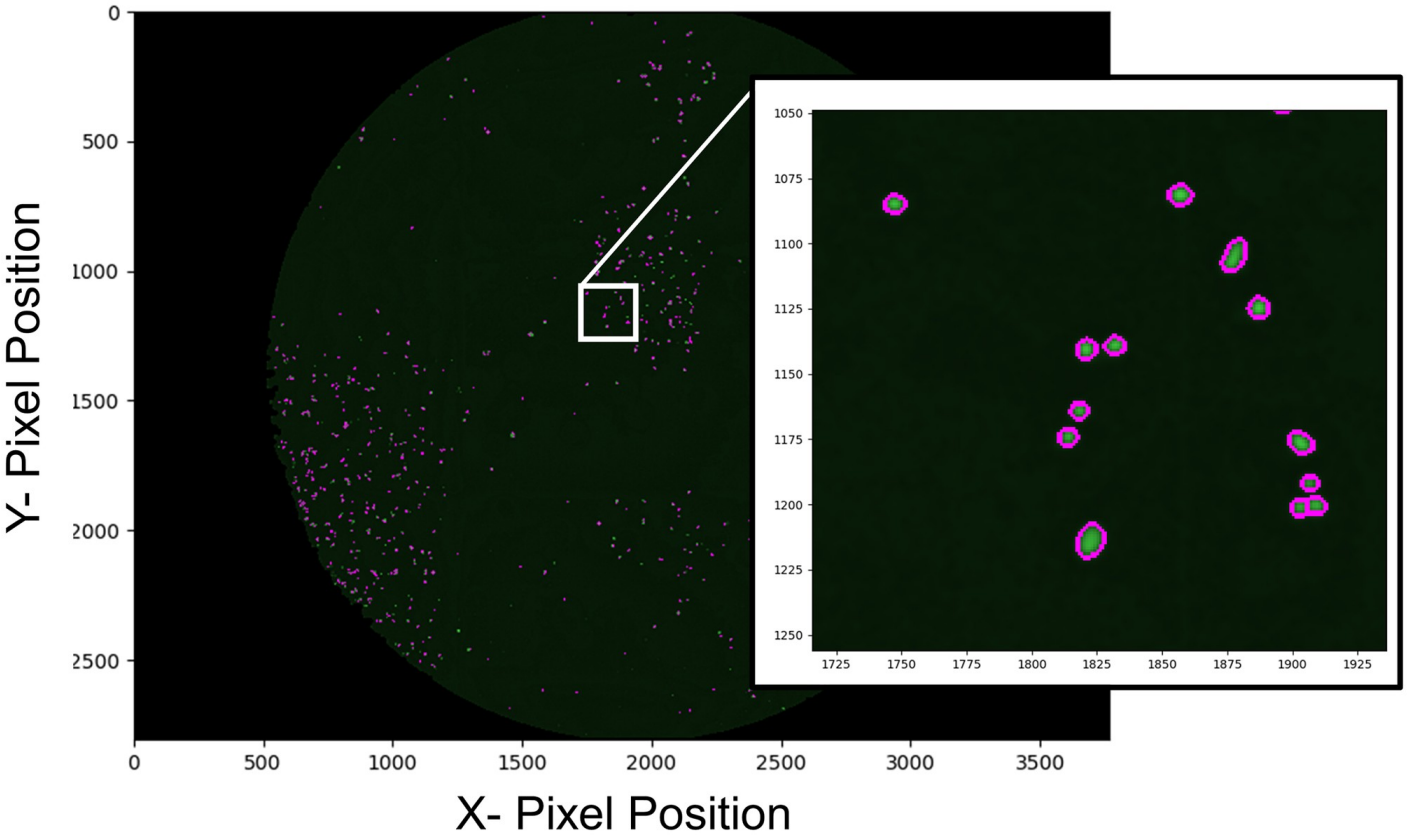
Final step: Cell counting. MCF-7 cells were processed via the optimized imaging pipeline. The final step outlines each cell nuclei and generates a merged image of the outlines(purple outlines) and H2B-GFP fluorescent nuclei (green). Inset is a magnification of the area outlined within the white box.

We tested several contrast adjustments in CellProfiler and Matlab to increase the visibility of cell nuclei. We found the log transform in Matlab performed the best (S1 Fig steps 7–8). Although this does require importing images into another software platform, the calculations were quick (taking only 1–2 minutes for 96 images) and provided images with readily detectable nuclei (S1 Fig steps 7–8). The resulting images were then imported back into CellProfiler, and the illumination function (described above) was applied. We found that after this log-transformation, all cells were readily identified by CellProfiler, which we confirmed by manual counting random images. After this step, we added a few standard clean-up processes to facilitate cell counting, such as masking the illumination corrected images, and applying a mild gaussian blur to remove artifacts and random noise (each step is described in S1 Fig). This combination of steps produced finely polished images containing distinct cell nuclei that CellProfiler could readily identify and count (Fig 8).

**Fig 9:**
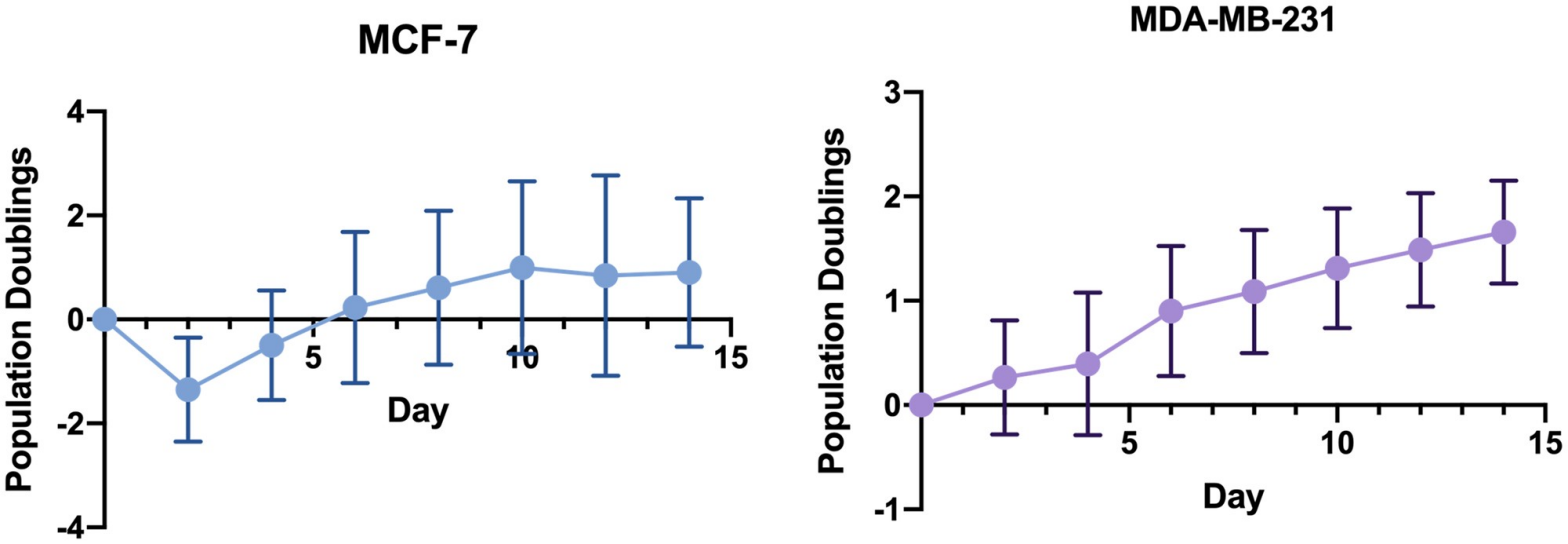
Assessment of cancer cell growth via image analysis pipeline. MCF-7 (left) and MBA-MD-231 (right) cells were seeded at low density (100 cells per well, 96-well plate). They were scanned every 2 days to quantify their growth. Images were processed via the developed pipeline, as described in methods. Data is expressed as population doublings. Mean ± SD (n = 12).

To demonstrate the cell counting pipeline, we’ve assessed the growth of MCF-7 and MDA-MB-231 breast cancer cells lines over a two-week period, imaging every two days (n = 12 wells each, Fig 9). All images were assembled into a single folder, and the pipeline was applied to generate growth curves for each cell type. The high number of replicates afforded by this approach reveals the phenotypic variation between wells, as well as between both cell types. Not unexpectedly, MDA-MB-231 cells—known for their fast doubling times—established growth almost immediately, whereas the MCF-7 cells exhibited a slower growth rate.

## Discussion

Using multi-well plates to culture cells can substantially increase statistical power of experiments by supporting addition of numerous biological replicates. However, with each added replicate comes an increased demand on time needed to maintain the culture, perform experiments, and analyze results. Establishing cell counts in each well—usually to assess cell proliferation—can be performed several ways, most of which require killing the cells (e.g., MTT assay) or disrupting growth (e.g., cell dissociation via trypsinization). Assessing cell counts by imaging has distinct advantages over these other methods. However, without an automated tool to count cells in acquired images, it becomes the most tedious method of them all.

The long-term goal of our laboratory is aimed at defining the cellular and biochemical microenvironment of tissues and tumors; and determining how cells coordinate and communicate to maintain tissue homeostasis. We have generated many different primary models and are interested in investigating consequences of cell-specific signaling. We therefore perform many co-culture assays in 96 well dishes and image them using our EVOS system. These scans create coherent images of each well, but analyzing these images manually (or semi-manually) was a long and tedious process. We therefore sought to develop an automated analysis pipeline (S1 Fig).

During development of the pipeline, we encountered many obstacles. Some of these related to how we cultured the cells, such as high background (Fig 2), debris and noise (Fig 3); whereas others were unique to the imaging process, such as Illumination and focus inconsistencies (Figs 4 & 5)—all of which interfered with our ability to accurately identify and count cells. During processing, we discovered that the EVOS software would often compress the well into an ovoid shape (Fig 6) and that the precise location of the wells varied in each image (Fig 7). Analysis of stitched images also presented a unique problem, in that the illumination artifact created a grid pattern in the composite image (Fig 1; S1 Fig, steps 9–10). We were able to overcome these problems and have outlined, in detail, an automatic analysis pipeline that can be used to quantify cell nuclei within tiled images. This was tested on images produced by the EVOS system, but we predict it can be easily adapted to images acquired by similar systems.

The current paper represents a series of considerations and suggestions for the use with the EVOS platform and high-throughput imaging and common issues that may be encountered. The current pipeline has already demonstrated applications beyond our original intent, and we continue to seek ways to further refine and advance its sensitivity to input data. We submit the current pipeline in hopes that it will assist others with similar applications and goals.

## Supporting information

S1 Fig Graphical overview of processing steps. The final processing pipeline contains three distinct processing groups: Cropping (top), Log transformation (middle), and Counting (bottom). The top and bottom groups of steps are performed in CellProfiler (red boxes), whereas the log transformation (middle blue box) is performed in Matlab. Cropping the images consists steps that open the image, converts the color image to grey and masks the grey image to remove areas exterior to the well. The second processing group performs a log contrast transformation of each pixel, which is essential to increase the cell nuclei signal relative to background. Finally, the third processing group contains steps that correct illumination variance, removes random noise, identifies cell nuclei, and overlays outlines of the identified nuclei onto the greyscale image. An annotate pipeline is available at https://cellprofiler.org/examples/published_pipelines. EVOS images were imported into CellProfiler 3 from their raw acquired state, and processed to convert the images to greyscale, then inverted and smoothened to identify the well as an object. Once identified, a mask of the well was created and applied to the greyscale image to remove everything outside of the well. To prevent attenuation of the cell nuclei signal that occurs during the illumination correction preprocessing step, a log contrast transform is applied (in Matlab) after masking the image. The resulting image sets are then imported back into CellProfiler 3 to a) remove illumination artifacts caused by the EVOS stitching algorithm, b) remove boarder pixels (erosion), and c) reduce noise within the images by smoothing with a gaussian blur. Finally, the processed images are analyzed for nuclei signals based on pixel intensity and diameter. Outlines of the identified nuclei are overlaid on the original input image and summarized into a .csv output file. Versions of all the analysis CellProfiler3 pipelines and Matlab code are available for download from: BreastCancerLab.com, http://breastcancerlab.com/evos-cell-counting-pipeline/.
(TIF)
