# Supplementary figures and images for: A high-throughput imaging and quantification pipeline for the EVOS imaging platform
Source: PLoS One. 2020 Aug 5;15(8):e0236397. doi: 10.1371/journal.pone.0236397 (PMC7406032; doi:10.1371/journal.pone.0236397)

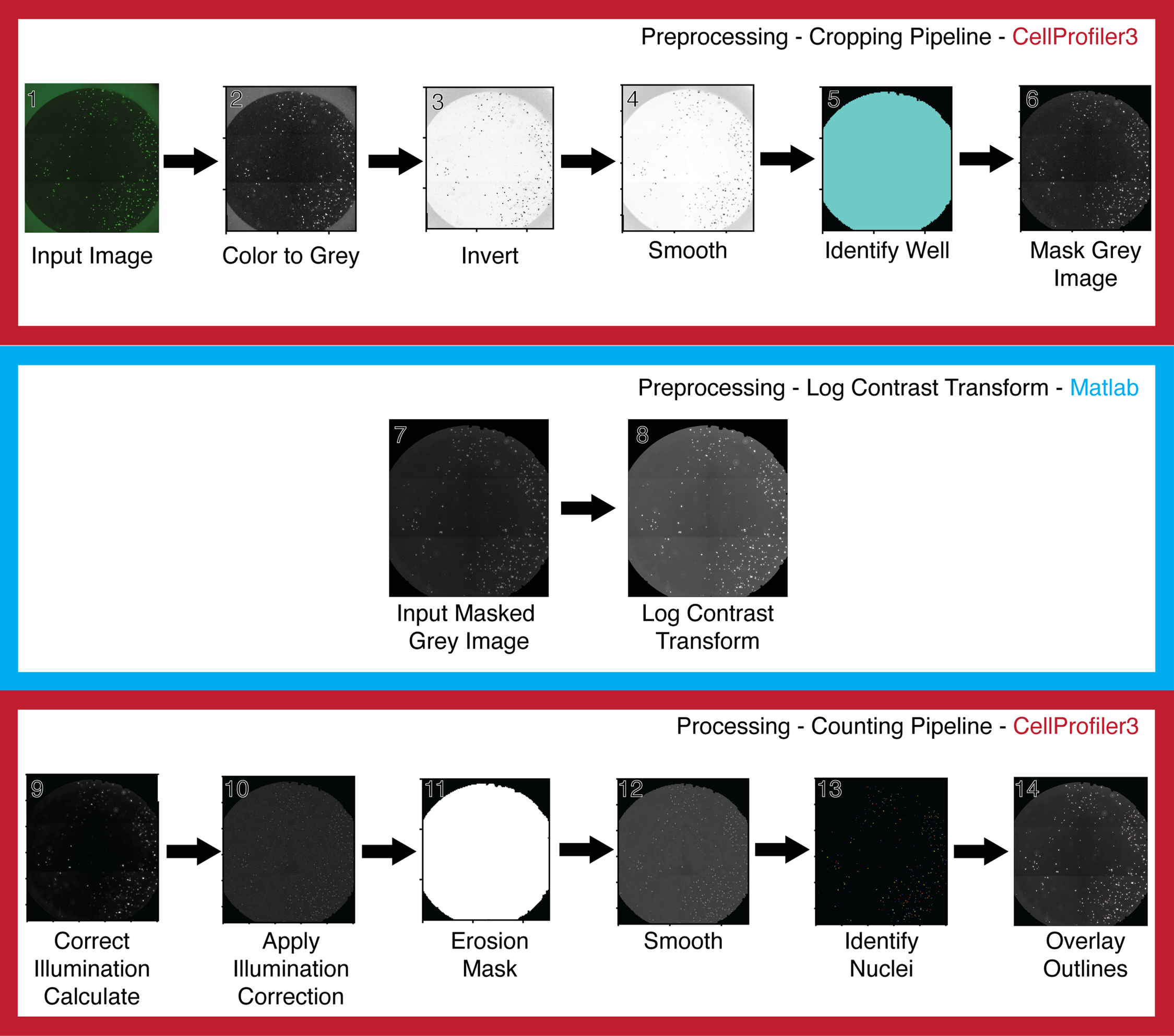

Supplement: S1 Fig — The final processing pipeline contains three distinct processing groups: Cropping (top), Log transformation (middle), and Counting (bottom). The top and bottom groups of steps are performed in CellProfiler (red boxes), whereas the log transformation (middle blue box) is performed in Matlab. Cropping the images consists steps that open the image, converts the color image to grey and masks the grey image to remove areas exterior to the well. The second processing group performs a log contrast transformation of each pixel, which is essential to increase the cell nuclei signal relative to background. Finally, the third processing group contains steps that correct illumination variance, removes random noise, identifies cell nuclei, and overlays outlines of the identified nuclei onto the greyscale image. An annotate pipeline is available at https://cellprofiler.org/examples/published_pipelines. EVOS images were imported into CellProfiler 3 from their raw acquired state, and processed to convert the images to greyscale, then inverted and smoothened to identify the well as an object. Once identified, a mask of the well was created and applied to the greyscale image to remove everything outside of the well. To prevent attenuation of the cell nuclei signal that occurs during the illumination correction preprocessing step, a log contrast transform is applied (in Matlab) after masking the image. The resulting image sets are then imported back into CellProfiler 3 to a) remove illumination artifacts caused by the EVOS stitching algorithm, b) remove boarder pixels (erosion), and c) reduce noise within the images by smoothing with a gaussian blur. Finally, the processed images are analyzed for nuclei signals based on pixel intensity and diameter. Outlines of the identified nuclei are overlaid on the original input image and summarized into a .csv output file. Versions of all the analysis CellProfiler3 pipelines and Matlab code are available for download from: BreastCancerLab.com, h [file pone.0236397.s001.tif]
